# A Review of the Neurosurgical Management of Brain Metastases During Pregnancy

**DOI:** 10.1017/cjn.2020.254

**Published:** 2021-09

**Authors:** Phileas J. Proskynitopoulos, Fred C. Lam, Sunjay Sharma, Brett C. Young, Yosef Laviv, Ekkehard M. Kasper

**Affiliations:** Department of Surgery, Division of Neurosurgery, Hamilton General Hospital, McMaster University, Hamilton, ON, Canada; Department of Surgery, Division of Neurosurgery, Tel Aviv; Department of Obstetrics and Gynecology, Beth Israel Deaconess Medical Center, Harvard Medical School, Boston, MA, USA

**Keywords:** Brain metastases, Pregnancy, Pregnancy-associated secondary tumors, Neurosurgery, Chemotherapy, Radiation therapy

## Abstract

**Objective:**

Patients with pregnancy-associated secondary brain tumors (PASBT) are challenging to manage. Because no guidelines for the management of such patients currently exist, we performed a systematic review of the literature using PRISMA guidelines with a discussion of management from a neurosurgeon’s perspective.

**Method:**

Systematic review of the literature using PRISMA guidelines from 1999 to 2018.

**Results:**

We identified 301 studies of which 16 publications (22 patients reporting 25 pregnancies, 20 deliveries, 5 early terminations) were suitable for final analysis. The most frequent primary cancers were breast (8/22, 36.36%), skin (6/22, 27.27%), and lung (5/22, 22.73%). Four patients (18.18%) had neurosurgical procedures during their pregnancies. Five patients (22.73%) received neurosurgical resection after their pregnancies. Nine patients (40.91%) received radiation therapy and seven patients (31.82%) received chemotherapy during pregnancy while seven patients (31.82%) received chemotherapy and radiation after pregnancy. There was 1 fetal death (5%) out of 20 healthy deliveries. Five pregnancies (20%) were terminated in the first trimester due to a need for urgent neurosurgical intervention.

**Conclusion:**

Management of PASBT remains a challenging issue. Maternal and fetal risks associated with surgical resection and teratogenicity due to adjuvant therapy should be discussed in the context of a multidisciplinary team. Timing of surgery and the use of systemic chemoradiation depends on the gestational age (GA) of the fetus, extent, and control of the mother’s primary and metastatic disease. Guidelines need to be established to help neuro-oncology teams safely and effectively manage this group of patients.

## RÉSUMÉ

**Étude de la prise en charge neurochirurgicale de métastases cérébrales détectées pendant la grossesse**.

**Objectif:**

La prise en charge de patientes enceintes chez qui l’on a diagnostiqué des tumeurs cérébrales secondaires n’est pas chose facile. Sachant qu'il n'existe actuellement aucune ligne directrice en matière de prise en charge, nous avons réalisé une revue systématique de littérature au moyen des lignes directrices PRISMA (*Preferred Reporting Items for Systematic Reviews and Meta-Analyses*) tout en proposant une discussion sur le sujet d’un point de vue neurochirurgical.

**Méthode:**

Une revue systématique de littérature au moyen des lignes directrices PRISMA, et ce, de 1999 à 2018.

**Résultats:**

Au total, nous avons identifié 301 études. De ce nombre, 16 d’entre elles ont pu se prêter à une analyse finale. À noter que ces 16 études incluaient 22 patientes ayant rapporté 25 grossesses, 20 accouchements et 5 interruptions de grossesse prématurées. Les cancers primitifs les plus fréquents étaient ceux du sein (8/22, soit 36,36 %), de la peau (6/22, soit 27,27 %) et du poumon (5/22, soit 22,73 %). Soulignons que 4 patientes (18,18 %) ont subi des interventions neurochirurgicales en cours de grossesse tandis que 5 d’entre elles (22,73 %) ont bénéficié de résection neurochirurgicale après leur accouchement. De plus, 9 patientes (40,91 %) ont reçu des traitements de radiothérapie, 7 d’entre elles (31,82 %) ont reçu des traitements de chimiothérapie en cours de grossesse et 7 autres (31,82 %) ont reçu à la fois des traitements de radiothérapie et de chimiothérapie à la suite de leur accouchement. Sur 20 accouchements de fœtus jugés en bonne santé, on a constaté un décès (5 %). Enfin, 5 grossesses, soit 20 %, ont dues être interrompues lors du premier trimestre en raison d’une intervention neurochirurgicale urgente.

**Conclusion:**

La prise en charge de patientes enceintes chez qui l’on a diagnostiqué des tumeurs cérébrales secondaires demeure un enjeu compliqué à aborder. Les risques associés à la résection chirurgicale et à la tératogénicité de la chimiothérapie adjuvante auxquels font face mères et fœtus devraient être discutés dans le cadre d’une équipe multidisciplinaire. Le moment choisi pour une intervention chirurgicale et l’utilisation systématique de chimio-radiation dépendent entre autres de l’âge gestationnel du fœtus mais aussi de l’étendue et du contrôle de la maladie métastatique primitive de la mère. Chose certaine, des lignes directrices devraient être établies en vue d’aider les équipes de neuro-oncologie à prendre en charge ces patientes de manière sûre et efficace.

## Introduction

There has been a trend toward delaying motherhood in developed countries, resulting in an average age of 26.3 years for mothers delivering their first-born child in the USA in 2014.^6^ Unfortunately, this delay in motherhood has led to an overlap with the period of increasing natural incidences of certain cancers. At present, the cumulative incidence of cancers in pregnant women has been estimated to be at around 1 in 1000–2000 pregnancies,^3^ further creating a unique and challenging clinical scenario of pregnancy-associated secondary brain tumors (PASBT). Among these, there has been an increased incidence of breast, melanoma, and lung cancer, all of which have propensities to metastasize to the brain.^35^

There are obvious risks to the fetus when treating expectant mothers diagnosed with cancer using chemoradiation. Beyond that, there are elevated anesthesia risks and other comorbidities associated with many non-obstetrics-related surgical procedures when performed during pregnancy.^12,14,28^ It is noteworthy that there are currently no guidelines regarding the neurosurgical treatment of PASBT. To address this void, we performed a systematic review of the literature with a focus on PASBT to coalesce common and unique challenges in this setting and to discuss key aspects in managing these patients from a neurosurgeon’s perspective.

## Materials and Methods

### Data Sources

A PRISMA-guided literature search in MEDLINE (PubMed) was conducted using the search phrases “brain”, “intracranial”, “intracerebral”, “cancer”, “metastasis”, “pregnancy”.

An additional search was conducted using Google Scholar to avoid missing articles that may have lacked one or more of the above search terms.

### Search Criteria

**Figure 1:**
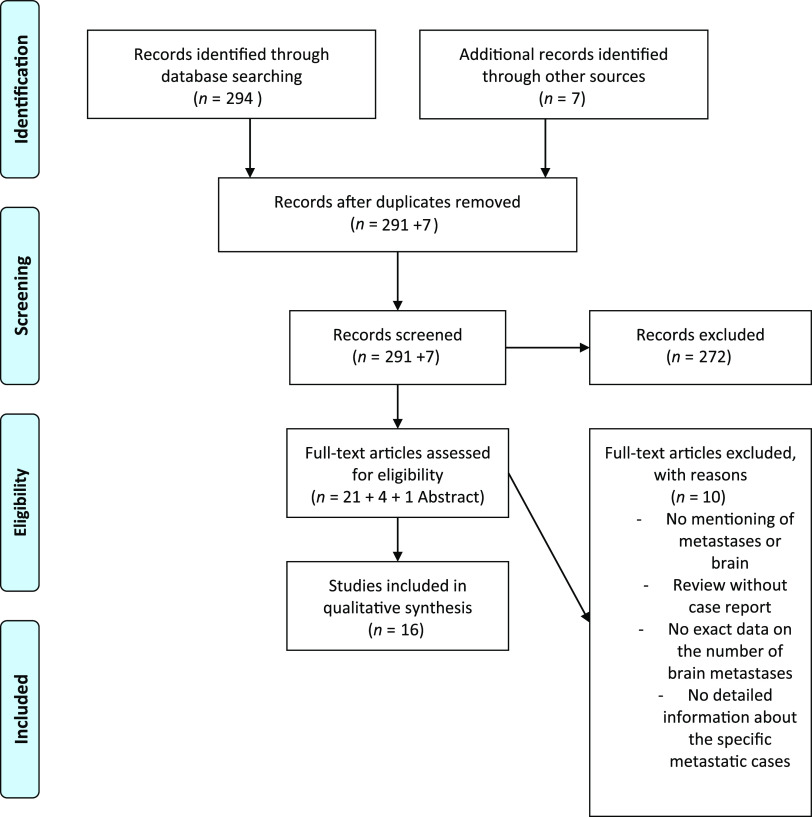
PRISMA 2009 flow diagram.

Studies had to meet the following criteria to be included in our analysis: (1) Published in the MRI era between January 1990 and August 2018; (2) English language; (3) Human subjects; and (4) Peer-reviewed publications. A PRISMA workflow diagram is provided in Figure 1.

### Data Extraction

The following information had to be available in selected articles and was extracted from all suitable publications identified in our PubMed search: (a) Classification of primary malignancy; (b) Description of brain metastases; (c) Diagnostic method and criteria for detection of brain metastases; (d) Information regarding pregnancy status and management, including outcome; (e) Description of details regarding treatment of brain metastases during pregnancy; (f) Age of the mother; (g) Gestational age (GA) of the fetus at diagnosis and delivery; (h) Method of delivery.

## Results

A total of 301 studies were retrieved after the application of our PRISMA algorithm (Figure 1). Of these, 16 fulfilled all inclusion criteria, and 2 studies presented more than 1 case. This resulted in a pooled cohort of 22 cases of PASBT, which we included in our final analysis. Individual cases and reports are summarized in detail in Table 1.

**Table 1:** Summary table of relevant cases of PASBT identified through our PRISMA search

Study	Patient age, GA at diagnosis	Primary cancer diagnosis	Location of brain metastasis	Timing and treatment for brain metastases	Pregnancy outcome	Maternal–fetal outcome
Magne et al. 2001^23^	38 years	Lung cancer	Right occipital lobe	Gross total resection ***during*** second pregnancy at 24 weeks GA with five sessions of post-op WBRT and maternal–fetal lead body shielding with thermoluminescent dosimeters.		
	First pregnancy, 18 weeks GA				First pregnancy: termination of pregnancy at 18 weeks GA.	Mother and infant are alive at end of the study.
	Second pregnancy, 24 weeks GA				Second pregnancy: C-section at 32–33 weeks GA.	Child displayed normal growth and development at the last follow-up at 3 years of age.
Yu et al. 2003^50^	40 years, 24 weeks GA	Malignant melanoma	Right occipital lobe	Single-dose GKRS ***during*** pregnancy at 25 weeks GA. Fetal radiation doses were assessed with phantom measurements before GKRS and using thermoluminescent dosimeters during GKRS.	C-section at 34 weeks GA following GKRS.	Both mother and infant did well and experienced no complications. Child development is considered normal at 14 months follow-up.
Pages et al. 2010^36^	28 years, 17 weeks GA	Superficial spreading melanoma	Unknown	Fotemustine ***during*** pregnancy at 17, 18, 19, 24, and 28 weeks GA; WBRT ***during*** pregnancy at 23 weeks GA.	Emergency C-section at 30 weeks GA.	Mother died 8 days after delivery. Infant is alive at 23-months follow-up.
	36 years, 24 weeks GA	Superficial spreading melanoma	Unknown	SRS ***during*** pregnancy at 24 weeks GA.	Emergency C-section at 32 weeks GA.	Mother died 2 months after delivery. Infant is alive at 2 months.
	34 years, 30 weeks GA	Superficial spreading melanoma	Unknown	Unknown	Emergency C-section at 31 weeks GA.	Mother died 3.5 months after treatment. Infant is alive at 60-months follow-up.
Goutham et al. 2011^13^	27 years, 9 weeks GA	Malignant melanoma	Tumor emboli to right and left temporal lobes causing malignant cerebral edema, hydrocephalus	Ventriculoperitoneal shunt for hydrocephalus ***after*** termination of pregnancy.	Termination of pregnancy at 9 weeks GA.	Mother succumbed to malignant cerebral edema.
			Spine mets at T3–T4, T8, and T10–T11	T4–T8 laminectomy and decompression ***after*** the termination of pregnancy.		
Mandrawa et al. 2011^24^	25 years, 28 weeks GA	Breast cancer	Left frontal cortex, right frontoparietal cortex, posterior fossa	Trastuzumab ***during*** pregnancy. Posterior fossa decompression with WBRT and SRS boost to tumor resection followed by lapatinib and capecitabine ***after*** delivery.	Forceps delivery at 37 weeks GA.	Mother and child are alive at the time of study termination.
Gupta et al. 2014^15^	24 years, 22 weeks GA	Breast cancer	Left thalamus and parietal cortex	Trastuzumab was held at the beginning of pregnancy. WBRT was administered ***during*** pregnancy at 22 weeks GA with external shielding followed by trastuzumab. Switched to capecitabine and lapatinib ***after*** pregnancy.	C-section at 38 weeks GA.	Infant is delivered with normal Apgar scores. Mother died from metastases 6 months postpartum. Infant is healthy at the last follow-up of 6 months.
Verheecke et al. 2014^47^	26 years, 17 weeks GA	Lung cancer	Two metastases, location unspecified	Radiotherapy with vinorelbine and cisplatin ***during*** pregnancy. Cisplatin and pemetrexed ***after*** pregnancy.	Termination of pregnancy of twins at 23 weeks GA due to oligohydramnios.	Mother died 12 months after primary diagnosis.
	25 years, 17 weeks GA	Wilms’ tumor	Unspecified	WBRT ***after*** pregnancy.	C-section at 30 weeks GA.	Mother and child are alive and is stable at 4 years of age.
	34 years, 28 weeks GA	Lung cancer	Unspecified	Radiotherapy, cisplatin, and teniposide ***after*** pregnancy.	C-section at 30 weeks GA.	Mother died 10 months after primary diagnosis. Child is alive and well at the age of 14 years.
	30 years, 26 weeks GA	Breast cancer	Unspecified	Radiotherapy ***during*** pregnancy.	C-section at 36 weeks GA.	Local relapse 1 month after delivery, stable disease at 2 years after diagnosis. Child is alive and well.
	41 years, 33 weeks GA	Melanoma	Unspecified	Cisplatin, decarbazine, and vinblastine ***during*** pregnancy. Radiotherapy ***after*** pregnancy.	C-section at 33 weeks GA.	Mother died 9 years and 9 months after primary diagnosis. Child is alive and well.
Vetter et al. 2014^48^	29 years, 25 weeks GA	Breast cancer	Multiple, largest 3.5 cm, cerebellar lesions with brainstem compression	Resection of cerebellar mass ***during*** pregnancy. Palliative WBRT with docetaxel, trastuzumab, and pertuzumab ***after*** pregnancy.	C-section at 30 weeks GA.	Mother and child are alive at the time of study termination.
Holzmann et al. 2015^17^	29 years, 26 weeks GA	Lung cancer	Multiple supratentorial and thoracic levels 2 and 3 spinal metastases	One cycle of docetaxel and carboplatin and radiotherapy to thoracic spine ***during*** pregnancy at 27 weeks GA. Gefitinib started 2 days ***after*** C-section.	C-section at 30 weeks GA.	Mother died at 17 months after primary diagnosis. Child is alive and well.
Okuda et al. 2016^34^	35 yo, 18 weeks GA	Recurrent breast cancer	Initial diagnosis: left temporal lobe	Surgical resection of temporal lobe metastasis ***after*** the delivery of her first pregnancy.	C-section of first pregnancy at 36 weeks GA.	The first child is alive and well at 5 years of age.
			Recurrence: multiple brain lesions	WBRT with adriamycin and cyclophosphamide ***after*** termination of her second pregnancy.	Termination of second pregnancy at 21 weeks GA.	No long-term follow-up of the mother was reported.
Sharma et al. 2016^42^	35 years, 9 weeks GA	Breast cancer	Right frontal lobe	Capecitabine, lapatinib, temozolomide, and CKRS ***during*** pregnancy at 9 weeks GA. Craniotomy for recurrent disease ***during*** pregnancy at 16 weeks GA. 5-FU, adriamycin, and cyclophosphamide for disease recurrence ***during*** pregnancy at 22 weeks GA. Repeat craniotomy ***during*** pregnancy at 27 weeks GA with discontinuation of chemotherapy. WBRT ***during*** pregnancy at 30 weeks GA.	C-section at 32 weeks GA.	Mother and child are alive and healthy at 6 weeks follow-up.
Huang et al. 2017^18^	37 years, 21 weeks GA	Thyroid cancer	Large scalp lesion with bony invasion	Surgical resection of scalp and skull lesion ***after*** pregnancy.	Delivered at term gestation.	Mother and child are alive and healthy at 5 months follow-up.
Kumagai et al. 2017^21^	37 years, 18 weeks GA	Large B-cell non-Hodgkin’s lymphoma	Multiple brain and spine lesions	Chemotherapy started ***during*** pregnancy at 18 weeks GA.	C-section at 33 weeks GA.	Mother died 23 days after C-section. Infant died at 18 days postpartum.
Paulsson et al. 2017^38^	42 years, 29 weeks GA	Breast cancer	Multiple lesions in frontal and temporal lobes and cerebellum	Craniotomy and gross total resection of frontal metastasis ***during*** pregnancy at 24 weeks GA. Post-op GKRS at 25 weeks GA with fetal dose estimation and risk assessments.	C-section at 36 weeks GA.	Mother and child are alive and well at 3.5 years follow-up.
Trinca et al. 2017^47^	35 years, 9 weeks GA	Breast cancer	Unspecified	WBRT, SRS, Temozolomide ***after*** early termination of pregnancy.	Early termination of first pregnancy. Second child was born 2 years after primary diagnosis.	Mother and child are alive and well at 4 years follow-up.
Wang et al. 2017^51^	19 years, 38 weeks GA	Alveolar soft tissue sarcoma	Left occipital and left cerebellar lesions	Emergency craniotomy for raised ICP and resection of cerebellar lesion ***after*** delivery.	C-section at 38 weeks GA.	Mother and child are alive and well at 10 months follow-up.

C-section = cesarean section; CKRS = CyberKnife Radiosurgery; GA = gestational age; GKRS = Gamma Knife Radiosurgery; ICP = intracranial pressure; SRS = stereotactic radiosurgery; WBRT = whole-brain radiotherapy; 5-FU = 5-Fluorouracil.

### Tumor Diagnoses

Eight of the 22 patients (36.36%) had breast cancer: Six with intraductal/infiltrative breast cancer and two with triple-negative breast cancer. Six patients (27.27%) presented with a primary skin tumor: three of those were classified as superficial spreading melanoma, and three as malignant melanomas. Five patients (22.73%) had lung cancer and the remaining three patients (13.64%) had various pathologies, including one Wilms’ tumor; one follicular thyroid carcinoma; and one primary mediastinal large B-cell non-Hodgkin’s lymphoma.

### Chemotherapy Only

One patient presenting with spine and brain metastases due to large B-cell non-Hodgkin’s lymphoma at 18 weeks GA was treated with chemotherapy alone *during* the pregnancy.^21^

### Radiation Therapy Only

Four patients (18.18%) received single modality radiotherapy for the treatment of their brain metastases: three patients received stereotactic radiosurgery (SRS) *during* their pregnancy at 24 weeks GA,^36^ 25 weeks GA^50^ and 26 weeks GA.^47^ One patient received whole-brain radiotherapy (WBRT) *after* her elective C-section at 30 weeks GA.^47^

### Neurosurgical Procedure Only

Three patients (13.64%) had neurosurgical interventions *after* their pregnancies: One patient had an insertion of a ventriculoperitoneal (VP) shunt after early termination of her pregnancy at 9 weeks GA^13^; Two patients had surgical resection of their tumors after successful term deliveries.^18,34^

### Chemoradiation Only

Seven patients (31.82%) received combination chemotherapy and radiation. Five patients received chemotherapy and radiation both *during* and *after* their pregnancies,^15,17,23,36,47^ while two patients received treatments *after* their pregnancies.^34,46^

### Combined Neurosurgical Intervention and Chemoradiation Therapy

Four patients had combined interventions: One patient had chemotherapy *during* her pregnancy followed by neurosurgical resection and chemoradiation after successful delivery of her child^24^; the second patient received surgical resection of her metastasis *during* pregnancy followed by WBRT and chemotherapy after successful delivery of her child^49^; the third patient received chemoradiation prior to neurosurgical resection followed by further resection at the time of recurrence and WBRT *during* her pregnancy with the successful delivery of a healthy fetus.^42^ Timing of the above interventions with maternal/fetal outcomes is summarized in Table 1.

## Discussion

The detection of brain metastasis during pregnancy is a rare occurrence. However, the risks surrounding the treatment of the mother’s primary tumor and metastatic disease have to be balanced against maintaining the health and viability of the fetus and those competing treatment goals pose significant challenges to the neurosurgeon.

It is estimated that 20% of cancer patients will develop brain metastases.^1,30^ Of these, lung (20–56%), breast (5–20%), and melanoma (7–16%) most commonly metastasize to the brain with the prevalence of renal cell carcinoma and colorectal cancer on the rise.^1,5^ Lung cancer is the most frequent to metastasize to the brain irrespective of gender, but breast cancer metastases are the most common occurrence in women.^1^ Risk of brain metastases also varies with age and the type of primary, with breast cancer brain metastases being reported as highest in younger patients (20–39 years of age), lung cancer brain metastases the highest in middle-aged patients (40–49 years of age), and melanoma, renal cell carcinoma, and colorectal cancer brain metastases highest in older patients (50–59 years of age).^5,17,19^ Interestingly, the increased risk of brain metastases in patients younger than 35 years of age was independent of cancer subtype, however, triple-negative or HER2-positive breast cancer subtypes were associated with a higher risk of brain metastases in patients older than 35 years of age.^1^ These epidemiologic findings highlight the need for neurosurgical guidelines for the management of patients with PASBT, as the neurological disease causes up to 25% mortality in patients with brain metastases.^31,44^

### Guiding Algorithms

#### Safe Dosing of Radiation Therapy for Brain Metastases During Pregnancy

In a general cancer patient population of male and female nonpregnant individuals, there is Level 1 evidence that surgery followed by WBRT is recommended as first-line treatment in all patients with single brain metastases with a favorable performance status and limited extracranial disease, as outlined in the 2019 guidelines put out by the Congress of Neurological Surgeons.^29^ However, SRS to the tumor bed has recently been shown to have equivalent survival outcomes and just slightly inferior local control rates, and can thus be used as an effective alternative to WBRT (median survival of SRS = 12.2 months vs. WBRT = 11.6 months).^27^ This may be an appealing treatment alternative in this setting to attenuate neurocognitive deficits associated with WBRT, especially poignant in young expectant mothers with well-controlled systemic disease.^8^ Modern shielding techniques can scatter radiation and shield the fetus from the adverse effects of WBRT, including mental retardation, organ malformations, and radiation-induced childhood malignancies.^14,23,37,50^

#### Timing of Neurosurgical Intervention During Pregnancy

Of the 11 cases requiring neurosurgical intervention, only 1 case required emergent craniotomy to alleviate increased intracranial pressure due to tumor mass effect. Both the mother and her fetus survived the surgery and the fetus was subsequently delivered via cesarean section at 38 weeks GA.^49^ Surgery should be delayed if possible until after the first trimester to minimize miscarriage risk, which can be as high as 10.5%.^10,11^ However, it is generally considered to be safe during the second and third trimesters with recommended dosimetry guidelines for safety.^14,38,42,50^

To compare our study results to a larger patient population, we turned to a retrospective cohort study of pregnancy-related hospitalizations between 1998 and 2009 gathered from the Nationwide Inpatient Sample (NISQIP), a database of nonfederal US hospitalizations. Here, a recent study identified 19.75 million pregnancy-associated admissions, 397 associated with malignant brain tumors, including 165 associated deliveries (44%).^45^ Patients with malignant tumors had more pregnancy complications including preterm labor, intrauterine growth restriction, and stillbirths. The rate of craniotomies for malignant tumors was 31.4% although the study did not differentiate between primary or metastatic brain malignancies. This was much lower than the 50% rate of craniotomies from our systematic review, however, this may be due to a lower rate of metastatic brain tumors in the former study. There was also a higher rate of C-sections amongst pregnant women with malignant tumors compared to the normal population, similar to the 65% of cases that required C-sections in our systematic review.

Our systematic review also identified one patient who required VP shunting for hydrocephalus.^13^ A retrospective series of 77 pregnancies in 37 women with VP shunts showed that rates of shunt revisions were 10 times higher during pregnancy or the following 6 months ensuing likely due to raised intra-abdominal pressures causing distal shunt failure.^7^ This had led some neurosurgeons to consider ventriculoatrial (VA) shunts for pregnant women, but VA shunts carry significant risks of arrhythmias, infection, valvular dysfunction, and high morbidity associated with revisions. The neurosurgeon should, therefore, closely monitor the pregnant patient for signs and symptoms of shunt failure in the peripartum and postpartum periods.^44^

#### Timing of Use of Antileptic Drugs During Pregnancy

Important to the neurosurgical management of patients with supratentorial tumors is the use of antiepileptic drugs (AEDs) to manage symptomatic lesions presenting with seizures. Older generation AEDs such as valproic acid and phenobarbital have been associated with increased rates of teratogenicity and congenital malformations (up to 9.3% and 5.5%, respectively) such as cardiac defects, cleft lip/palate, neural tube defects, and dysmorphic syndromes.^16^ However, newer agents such as lamotrigine and levetiracetam (currently, the most commonly used AED in neurosurgery) have improved safety profiles for use in pregnancy with much lower reported rates of congenital malformations between 0.7% and 2.4%.^16,25^ Doses of AEDs should be tapered to the minimally effective dose to achieve seizure control and used for the shortest period of time possible during pregnancy.^16^

#### Surgical Considerations of the Pregnant Patient During Neurosurgical Procedures

Unique challenges exist during the positioning of the patient for non-obstetric surgeries during pregnancy. Prevention of positioning injuries to mother and fetus needs to be observed and the operating table should be centered and parallel to the longest wall in the room to minimize the need for relocation prior to the patient entering the operating room.^4^ When possible, the patient should be maintained in the left lateral recumbent position using a positioning wedge to avoid a flat supine position once transferred onto the operating table with attention to the application of non-constricting safety straps to not harm, yet prevent the patient from falling off the table.^2^ A uterine displacing wedge or chests rolls should be used to reduce pressure from the pregnant uterus on the vena cava and straps across the chest are discouraged to avoid restricting respiratory effectiveness.^9^ Pressure points on the mother’s body should be well padded to prevent ulcers and peripheral neuropathies during surgery. Hypothermia should be carefully avoided (e.g. with the use of a warming blanket). Intraoperative electronic fetal monitoring past 26 weeks GA may be applied if the fetus is viable, if the monitor does not physically interfere with the procedure, if an obstetrician is available with prior consent obtained to perform an emergency C-section for fetal distress, and if the type of surgery is safe for the interruption to perform an emergency delivery if warranted.^4,40^

#### Anesthetic Considerations for the Pregnant Patient

Special anesthetic considerations are also required for non-obstetric procedures during pregnancy in order to avoid the complications of fetal loss, premature labor, and delivery. Cardiopulmonary changes in maternal physiology during pregnancy can lead to unintended general hypotension, difficult laryngeal intubation with potential airway edema, hypoxemia, and increased uterine irritability leading to preterm labor.^32^ The mother should be informed regarding the low risk of teratogenicity associated with anesthetic agents and a neonatal team should be available and present should premature labor be anticipated. Regional anesthesia is preferred over general anesthesia where it is feasible to minimize drug exposure to the fetal and allow the mother to maintain her own airway, however, there is a lack of evidence supporting these claims in the form of randomized clinical trials.

In the case of neurosurgical procedures, general anesthesia can rarely be avoided.^33^ Maintenance of P_CO2_ during the surgery can also be challenging as ventilation should be aimed to keep levels within the normal range of pregnancy, however, hyperventilation may be required to decrease central vasodilation as a means of lowering intracranial pressures during tumor resection. Extubation should be performed with a full anesthetic reversal and preferably in the lateral position to avoid aspiration.^41^

### Suggested Algorithm for the Treatment of Brain Metastases in the Pregnant Patient

We recently published a case series reviewing the surgical management of 104 meningioma patients during pregnancy and proposed an algorithm dividing the date of diagnosis with GA of the fetus for the surgical management of these patients into two groups.^22^ Using a similar algorithm based on studies in our systematic review and current literature, we propose dividing patients with PASBT into three groups in order to guide the timing of neurosurgical interventions:

1. Group A – Patients at ≤ 13 weeks GA
  - Surgery and/or systemic chemotherapy or radiotherapy should be delayed past the first and second trimesters if possible to prevent teratogenic effects of anesthetic drugs, chemotherapy, and radiation, as well as premature labor caused by anesthesia-induced hypotension to the placenta and uterus.
  - If patients require radiation therapy, SRS is preferred over WBRT to reduce long-term neurocognitive dysfunction to the mother and congenital malformations to the fetus. Body shielding should be used to minimize radiation exposure to the fetus.
  - If surgery and/or chemoradiation cannot be avoided, then a frank discussion should be had with the patient explaining adverse effects to the fetus and options including early termination of pregnancy.
  - Any practical aspects of survival intervention (positioning/pinning) are usually not compromised yet in this patient subgroup, as the pregnant uterus remains small and patients can even be positioned prone without significant concerns.
2. Group B – Patients between 13 and 26 weeks GA
  - The same algorithm as in Group A can be applied to Group B patients but if the patient is diagnosed or becomes symptomatic at around 26 weeks GA, the option of C-section to deliver the fetus with support in a neonatal intensive care unit should be presented to the patient prior to treatment for her symptomatic brain metastases. Here, and only if circumstances allow, the goal for initial treatment would remain to bring the baby as close to 30 weeks of GA as possible, since the relative risk reduction of infant mortality per week is greatest before that date.^43^
  - If surgical intervention is required, the left lateral decubitus or supine position is the preferred way to perform surgery at this stage.
3. Group C – Patients at ≥ 26 weeks GA
  - Symptomatic patients requiring surgery and/or chemoradiation should be presented with the delivery of their fetus via C-section or forceps-assisted delivery (to avoid raised intracranial pressures that may be associated with straining during labor) prior to their neurosurgical intervention.
  - Late into the third trimester, the teratogenic effects of chemotherapy and radiation exposure are much lessened and may allow for treatment of the mother without needing to deliver the fetus but risks need to be discussed with the mother.
  - As above, if surgical intervention is required, the left lateral decubitus or supine position is the preferred way to perform surgery at this stage.

Finally, current advances in targeted therapies for the treatment of breast, lung, and melanoma cancers may dramatically change the way we approach the treatment of PASBT in the future. While there remains a paucity of information regarding the long-term teratogenicity of targeted therapies, the HER2-neu receptor antagonist trastuzumab has been used for the treatment of breast cancer during pregnancy.^24^ The BRAF inhibitor dabrafenib is classified as a category D agent and therefore contraindicated for the treatment of metastatic melanoma during pregnancy.^26^ Similarly, there is a lack of data pertaining to fetal safety for the EGFR inhibitor erlotinib, currently, the standard of care treatment for EGFR-mutated lung cancer in the USA,^20^ hence conventional standard chemotherapy remains recommended in the treatment of pregnant patients with lung cancer. We eagerly await longitudinal data using immunotherapies and other novel modalities for the treatment of brain metastases, which may be added to increase the neurosurgeon’s armamentarium in managing PABST.^1^

## Conclusion

In conclusion, neurosurgical management of PASBT is a challenging problem and should involve a multidisciplinary team of neuro-oncologists, anesthesiologists, obstetricians, neonatologists, and ethicists in order to fully inform the mother of the risks to herself and her unborn fetus. The increasing ability of systemic targeted therapies to effectively control a patient’s primary disease combined with research into understanding the process of brain metastasis formation will hopefully lead to novel therapies, which are both effective and safe for the health and viability of the mother and fetus.
